# Pure red cell aplasia as first manifestation of splenic marginal zone lymphoma-successful treatment with rituximab: a case report

**DOI:** 10.4076/1757-1626-2-6913

**Published:** 2009-08-18

**Authors:** Athanasios Anastasiadis, Dimitrios Margaritis, Ioannis Kotsianidis, Emmanouil Spanoudakis, Anna Christoforidou, Ioannis Kostopoulos, Constantinos Tsatalas

**Affiliations:** 1 Department of Haematology, Democritus University of Thrace Medical School, University Hospital of AlexandroupolisAlexandroupolisGreece; 2 Department of Pathology, Aristotle University Medical SchoolThessalonikiGreece

## Abstract

**Introduction:**

Acquired pure red cell aplasia is a rare disorder, usually appearing secondary to various pathologic conditions such as thymoma, systemic autoimmune diseases or in the course of lymphomas. Conventional treatment consists of immunosuppression with corticosteroids, antithymocyte globulin or cyclosporin-A.

**Case presentation:**

8 weekly courses of rituximab were administered to a patient who presented with pure red cell aplasia secondary to newly diagnosed splenic marginal zone lymphoma. Transfusion independence was achieved after the 6^th^ course, and pure red cell aplasia receded completely with therapy.

**Conclusion:**

Pure red cell aplasia may ensue early in the course of splenic marginal zone lymphoma and other low grade lymphomas. Rituximab is a safe and effective alternative treatment for pure red cell aplasia secondary to lymphoproliferative disorders.

## Introduction

Acquired pure red cell aplasia (PRCA) is characterized by normochromic normocytic anemia, reticulocytopenia and a marked reduction of bone marrow erythroblasts (<5%), without any defects in the white blood cell and megakaryocytic lineages [1-5]. Most cases are secondary to various systemic disorders, lymphomas comprising a major, yet heterogeneous group of primary causes. There are currently no specific treatment guidelines for PRCA, though proposals have been made.

## Case presentation

A 67-year-old Greek man was referred to our department because of leucopenia, marked anemia and splenomegaly. Progressively worsening anemia-related symptoms had started 8 weeks prior to admission. The patient also reported night sweats, but neither fever nor weight loss. Physical examination revealed pallor and moderate splenomegaly (5 cm below the left costal margin). The liver and lymph nodes were not palpable. The rest of the physical examination was normal.

**Figure 1.**
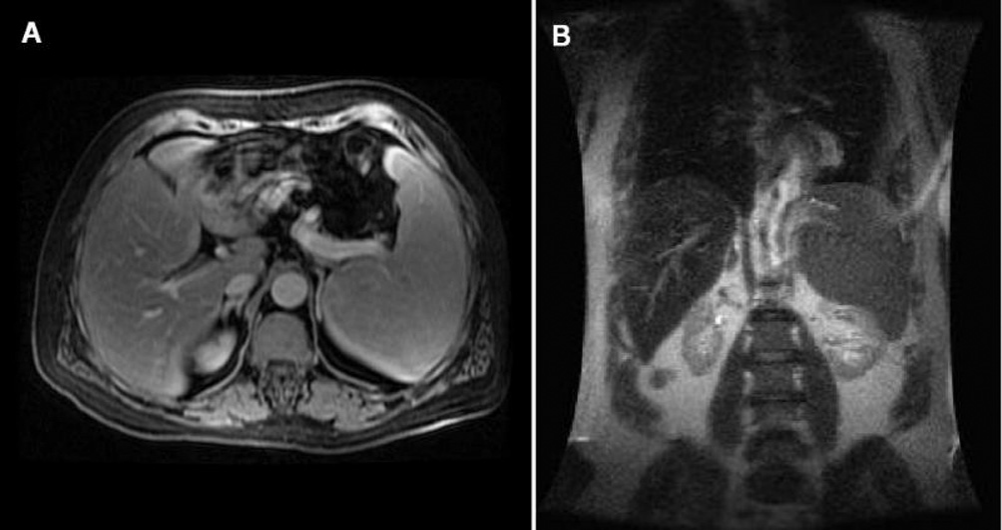
Axial **(A)** and coronal **(B)** view of the patient’s abdominal MRI. Splenomegaly is evident. Spleen size was calculated 18 cm × 15 cm × 11 cm. No focal lesions or hilar lymphadenopathy were detected.

Blood tests showed profound anemia and moderate neutropenia (Hct: 19.5%, MCV: 80fl, WBC: 2730/mm^3^, PMN: 1460/mm^3^, PLT: 249000/mm^3^). Serum ferritin was normal and reticulocyte count was 0.23%. Other abnormal tests included an elevated fasting serum glucose level (219 mg/dl), a prolonged partial thromboplastine time of 68.1 sec (norm. 26-35 sec) that was not corrected after 1:1 dilution with normal plasma, and an extremely high erythropoetin level of 409.4 U/ml (norm. 4-24 U/ml). Subsequent clotting factor assays showed the existence of a lupus anticoagulant. Serum electrophoresis and immunofixation did not reveal existence of a monoclonal paraprotein. Serology was negative for autoimmune disorders and viral infections (HBV, HCV, HIV and CMV). CT scan did not reveal any lymphadenopathy in the thorax, abdomen and pelvis, whereas CT and MRI of the upper abdomen showed diffuse splenomegaly without any focal lesions in the splenic parenchyma (Figure 1).

**Figure 2.**
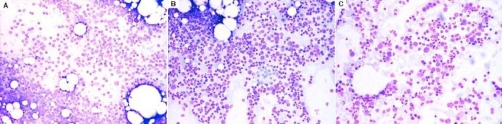
Bone marrow aspirates at various stages of treatment. **(A)** At diagnosis only sporadic proerythroblasts are visible. Aspirate consists mainly of the lymphoid infiltrate and a few neutrophils, plasma cells and mast cells. **(B)** After completion of treatment with rituximab erythroid series has recovered, a left shift evident. Lymphoid cells are still abundant. **(C)** One year after splenectomy erythroblasts in all maturation stages prevail. Lymphoid cells are sparse (<5%, confirmed by trephine biopsy).

A bone marrow aspirate and trephine biopsy were further obtained, revealing increased cellularity and a marked reduction and maturation arrest of the erythroid lineage in the proerythroblast stage. A lymphocyte infiltrate was also found, consisting of small, mature lymphocytes without villi (Figure 2A) and with paratrabecular, pericapillary and intrasinusoidal distribution in the marrow. Erythroid lineage comprised 4% and lymphocytes 60% of total bone marrow cells. Lymphocytes were CD20+, CD19+, CD22+, CD5-, CD10-, CD103-, CD23-, sIgM+ by immunohistochemistry and flow cytometry. No peripheral blood involvement was detected by flow cytometry. Additional serum testing for Parvovirus B19 was negative. Initial diagnosis was pure red cell aplasia secondary to low-grade non-Hodgkin's lymphoma.

The patient was treated with 8 weekly courses of rituximab at a dose of 375 mg/kg. Reticulocyte count rose to 5.3% after the 5^th^ cycle and he achieved transfusion independence after the 6^th^ cycle of treatment, having been transfused with a total number of 13 units of packed red cells since the day of admission. He was subsequently subjected to splenectomy. Biopsy of the spleen confirmed the diagnosis of splenic marginal zone lymphoma (SMZL). He remains alive and well since then, having achieved both complete remission of his PRCA (Figure 2B and 2C) and a very good partial remission of his SMZL, with a median hematocrit of 43% and a marrow infiltrate of less than 5% at subsequent marrow testing (Figure 2C).

## Discussion

PRCA is a rare disorder, defined as normochromic normocytic anemia, reticulocytopenia of <1% and marked reduction (<5%) or absence of erythroblasts in the bone marrow, without any abnormalities in the white blood cell and megakaryocytic lineages [1-5]. It is seldom idiopathic, the usual underlying cause being any of a variety of systemic disorders. The list includes thymoma, collagen vascular diseases, lymphoproliferative disorders, solid tumors, infections, severe renal failure, medications, pregnancy, severe nutritional deficiencies and other, rarer causes [1-5]. Possible pathogenetic mechanisms for its development include anti-erythropoetin antibody formation, T-cell mediated disruption of erythroblast maturation or NK-cell mediated cytotoxicity against the erythroblasts [3,5,6]. B-chronic lymphocytic leukemia (B-CLL) is the commonest of lymphomas associated with PRCA in its course (1-6% of all cases of B-CLL). T-cell large granular lymphocytic leukemia (T-LGL) is another common cause. Both diseases are known to cause immune dysregulation, autoimmune phenomena being their major features.

Splenic marginal zone lymphoma is an uncommon lymphoproliferative disorder, comprising 1% of all lymphoid neoplasms. The malignant cell immunophenotype is not distinctive and sometimes diagnosis is one of exclusion [6]. It usually presents with splenomegaly, lymphadenopathy limited to the splenic hilum and bone marrow infiltration. The liver and peripheral blood may be involved [6]. In one-third of cases a monoclonal IgM paraprotein is detectable and autoimmune phenomena like autoimmune hemolytic anemia, lupus anticoagulant and other acquired inhibitors to clotting factors have been described [6]. PRCA secondary to SMZL has been reported in the literature in only two cases so far [7,8], only one of whom was treated with rituximab [7]. SMZL is treated by splenectomy, a procedure aiding not only in debulking disease burden but also in reducing marrow infiltration. Recent reports have demonstrated the effectiveness of rituximab in patients for whom splenectomy is considered hazardous [9-11].

Treatment of PRCA consists of various types of immunosuppressive drugs, the most popular of which is corticosteroids. Other regimens include cyclosporine-A, antithymocyte globulin and azathioprine [12]. However, sporadic reports on the use of rituximab for PRCA secondary to B-cell lymphoid neoplasms, mainly B-CLL [13,14], are slowly emerging, adding a promising alternative to the current armamentarium. Although studies with more than four patients are lacking due to the rarity of the disorder, all reports up to date have shown excellent results.

In our patient's case, corticosteroids were avoided because of his newly diagnosed diabetes mellitus. Decision for rituximab administration was taken on the basis of its efficacy against both PRCA and SMZL. Although treatment resulted in remission of PRCA and a substantial reduction of marrow infiltration by the SMZL, splenectomy was finally decided because of the persistence of splenomegaly six months after discontinuation of treatment with rituximab.

## Conclusion

Although rare, PRCA must be considered as a possible cause of severe anemia in patients with lymphoproliferative disorders, even at diagnosis. For such cases rituximab appears to be a safe and effective treatment alternative to other immunosuppressive drugs.

## Abbreviations

B-CLL: B-chronic lymphocytic leukemia
CMV: cytomegalovirus
HBV: Hepatitis B Virus
HCV: Hepatitis C Virus
HIV: Human Immunodeficiency Virus
PRCA: pure red cell aplasia
SMZL: splenic marginal zone lymphoma
T-LGL: T-cell large granular lymphocytic leukemia
